# Multi‐Spatial Voxel‐Scale Modulation of Acupuncture on Abnormal Brain Activity in Migraine Patients Without Aura: A Randomized Study Neuroimaging Trial

**DOI:** 10.1002/brb3.70536

**Published:** 2025-05-08

**Authors:** Chaorong Xie, Zhiyang Zhang, Yutong Zhang, Xixiu Ni, Yang Yu, Xiaoyu Gao, Mingsheng Sun, Xiao Wang, Ling Zhao

**Affiliations:** ^1^ The Acupuncture and Tuina School Chengdu University of Traditional Chinese Medicine Chengdu Sichuan China; ^2^ The Third people's hospital of Chengdu Chengdu Sichuan China

**Keywords:** acupuncture, brain activity, multi‐spatial voxel‐scale, MwoA

## Abstract

**Background:**

The role of acupuncture in treating migraine has been widely recognized, but the systematic, comprehensive and, multi‐spatial voxel‐scale mechanism of brain function changes is still unclear.

**Objective:**

Resting‐state functional magnetic resonance imaging (rs‐fMRI) was used to investigate the modulatory effect of acupuncture on brain activity in patients with migraine without aura (MwoA) at different spatial voxel scales.

**Methods:**

A total of 64 patients with MwoA were randomized into true acupuncture (TA) and sham acupuncture (SA) groups. MwoA patients received TA or SA three times a week for four weeks, a total of 12 sessions. A clinical symptoms assessment and rs‐fMRI scans were evaluated before and after four weeks of treatment. Amplitude of low frequency fluctuation (ALFF) and fractional ALFF (fALFF), regional homogeneity (ReHo), and degree centrality (DC) were used to evaluate the spontaneous activity, activity coherence and connectivity importance of brain function at the single voxel, local voxel, and global voxel scales, respectively.

**Results:**

The clinical symptoms of both groups were improved compared with baseline. There were significant differences between the TA group and the SA group in migraine frequency, days and pain intensity. The neuroimaging data suggest that TA modulates a broader and more significant brain neural activity than SA. TA modulates the neural activity of the default mode network (DMN), visual network (VN), and sensorimotor network (SMN) at the single voxel scale, local voxel scale and global voxel scale, and these changes are correlated with the improvement of the migraine and quality of life.

**Conclusions:**

TA could exert therapeutic effects at different spatial voxel scales by modulating the DMN, VN, and SMN, which may be the neuroimaging mechanism of acupuncture for MwoA.

## Graphical abstract

Acupuncture treats migraine without aura by modulating the default mode network, visual network, and sensorimotor network at different spatial voxel scales.

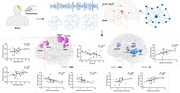

## 1 Introduction

Migraine is a common neurological disorder characterized by recurrent headache attacks accompanied by nausea, vomiting, photophobia, or phonophobia (“Headache Classification Committee of the International Headache Society (IHS) The International Classification of Headache Disorders, 3rd Edition,” 2018). Epidemiology shows that migraine affects approximately 1 billion people worldwide (Ashina et al. 2021). The 2019 global burden of the disease shows that migraine is ranked as the second leading cause of disability worldwide (Stovner et al. 2022), accounting for 5.6% of the global burden of disease (Ashina et al. 2021). Migraine has become a serious public health problem in society because of its repetition (Ashina 2020; Terwindt et al. 2000). The exact mechanism of migraine is currently unknown, and increasing studies have shown that migraine is associated with changes in central nervous system (CNS) activity (Ferrari et al. 2022; Messina et al. 2022, 2023). In the process of disease development, the default mode network (DMN), visual network (VN), salience network (SN), sensorimotor network (SMN), and other networks show abnormal functional activity (Chong et al. 2019; Messina et al. 2022; Schramm et al. 2023). At present, the first‐line treatment for mild‐to‐moderate acute migraine is primarily nonsteroidal anti‐inflammatory drugs (Ashina 2020; Ferrari et al. 2022), however, some patients have significant adverse reactions and irregular medication, leading to medication overuse headache (P.‐K. Chen and Wang 2019). Therefore, physicians began to seek other nonpharmacologic treatments as an effective supplement to pharmacologic therapy.

Acupuncture, as part of complementary and alternative medicine, has been proven in several studies to be safe and effective for the treatment of migraine (Y. Li et al. 2012; Xu et al. 2020; Zhao et al. 2017), and the long‐term prophylaxis effects of acupuncture may be at least as effective as preventive medications(Linde et al. 2016). Acupuncture has been found to reduce chronic pain by modulating various brain networks (Yu et al. 2022). The analgesic effect of acupuncture is a multilevel and multifactor compound therapeutic method, which mainly acts by integrating pulses from the pain regions and acupoints at different levels in the CNS (Qiao et al. 2020). In migraine, multiple pain‐modulating systems have been observed to be involved in the modulatory effects of acupuncture on abnormal brain activity and brain networks (C. Li et al. 2023; Z. Li et al. 2017; Ma et al. 2021; Raichle 2015; Zhao et al. 2014). These findings from neuroimaging studies have supported the central modulatory effect of acupuncture on migraine, but the mechanisms underlying the multispatial, voxelscale modulation of brain function remain unclear.

In rs‐fMRI, the amplitude of low frequency fluctuation (ALFF) is an important indicator to calculate the intensity of brain neural activity (Zhang et al. 2019). Fractional ALFF (fALFF)fALFF is considered to be an improvement of ALFF detection (Zou et al. 2008) and reduces the influence of ventricular and blood flow noise on ALFF detection (Zuo et al. 2010). Regional homogeneity (ReHo) assesses the similarity or synchrony of the time series between a particular voxel and its neighbors (Y.‐F. Zang et al. 2007). Degree centrality (DC) can calculate the number of connections between the brain area and other brain areas, and then reflect the functional importance and influence degree of the brain region in the whole‐brain network (Zuo et al. 2012). These four voxel‐based indicators reflect brain functional features from different scales and show a progressive and complementary relationship, which can identify the abnormalities of brain areas more comprehensively and sensitively. Therefore, it is appropriate to synthesize these indicators to explore the multi‐spatial, voxel‐scale modulation mechanisms of acupuncture on the CNS in migraine patients.

In this study, a randomized controlled trial and rs‐fMRI ALFF, fALFF, ReHo, and DC were used to compare the modulatory effects on brain function at single‐voxel, local‐voxel, and global‐voxel scales of migraine without aura (MwoA) between true acupuncture (TA) and sham acupuncture (SA). In addition, correlation analyses were performed to investigate possible correlations between clinical variables and changes in brain regions. We hypothesized that TA could modulate the default network and pain‐related abnormal brain network activity from different scales. This study is expected to deepen the understanding of the role of acupuncture in the central modulation of MwoA.

## 2 Methods

In this study, MwoA patients were recruited and screened from the hospital of Chengdu university of TCM and the third affiliated hospital of Chengdu university of TCM (Pidu district TCM hospital), and their clinical data and fMRI data were collected. This study was registered in the Chinese clinical trial registry (ChiCTR) (clinical trial registration: ChiCTR2000032308) and reviewed by the ethics committee of the Hospital of Chengdu university of TCM (ethical approval number: 2020KL‐003). The recruitment process began in May 2020 and continued through September 2022.

### 2.1 Participants

The diagnostic criteria of MwoA were according to the International Classification of Headache Disorders, 3rd edition (ICHD ‐ 3) diagnostic criteria of MwoA formulated by the IHS) in 2018 (“Headache Classification Committee of the International Headache Society (IHS) The International Classification of Headache Disorders, 3rd Edition,” 2018). The inclusion criteria were as follows: (1) age of 18–55 years old (onset age less than 50 years old), both sexes, ≥6 years of education; (2) meet the diagnostic criteria of MwoA of ICHD ‐ 3; (3) the number of attacks per month in the recent three months was greater than or equal to two times, and the number of headache days was less than 15 days per month; (4) baseline headache was defined as moderate headache (mean visual analog scale [VAS] 3–7 cm); and (5) informed consent was obtained from patients.

Exclusion criteria were as follows: (1) combined with other primary headache or headache of unknown diagnosis; (2) serious primary diseases of the cardiovascular and cerebrovascular, liver, kidney, hematopoietic system, and other organic lesions; (3) those with a history of head trauma, mental, or intellectual disability, who could not cooperate with the questionnaire; (4) received acupuncture or other preventive treatment within the past four weeks; (5) patients with metal implants, claustrophobia, or other contraindications to MRI examination; (6) severe brain anatomical asymmetry or definite lesions on MRI scan; and (7) participated in similar research within the past three months. Detailed inclusion and exclusion criteria can be found in the published protocol (J. Chen et al. 2022).

### 2.2 Study Design

In this trial, MwoA patients were observed for a total of eight weeks, of which a baseline period of the first four weeks checked whether MwoA patients were eligible, could correctly fill out the headache diary, and were willing to participate in the study. Statistical package for the social science statistics version 26.0 (SPSS 26.0; IBM Corp., Armonk, NY, USA) was used to generate a random number table of 64 patients, and patients who met the inclusion criteria were randomly divided into the TA group and the SA group in a 1:1 ratio. The participants in the TA and SA groups were blinded, and the acupuncturist could not perform the blinded treatment due to the particularity of the acupuncture process. The evaluators of clinical efficacy indicators were not involved in acupuncture operation, and the three‐separation principles of researchers, operators, and statisticians were implemented. In addition, each MwoA patient underwent fMRI scans before and after treatment.

### 2.3 Intervention

**FIGURE 1:**
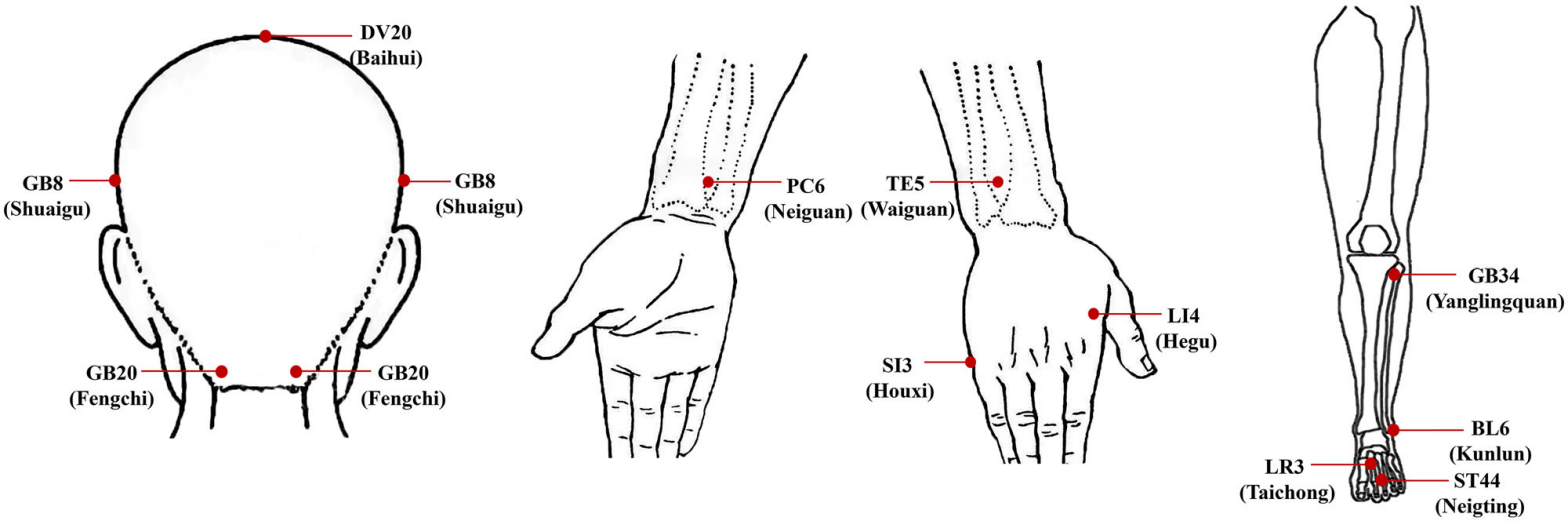
Acupoints locations. Abbreviation: BL, bladder meridian of foot sun; GV, governor meridian; GB, gallbladder meridian of foot shaoyang; LI, large intestine meridian of hand yangming; LR, liver meridian of foot Jueyin; PC, pericardium meridian of hand jueyin; SI, small intestine meridian of hand sun; ST, stomach meridian of foot yangming; TE, triple energizer meridian of hand shaoyang.

Patients in the TA and SA groups received a total of 12 acupuncture treatments over four consecutive weeks, with one treatment every other day, 3 times per week, each lasting 30 min. We selected the five acupoints for each treatment based on the results of previous studies (Zhao et al. 2017) and clinical expert consensus meetings (Figure 1). Baihui (GV20), Fengchi (GB20), and Shuaigu (GB8) were the three mandatory acupoints, and the other two acupoints were selected according to the meridian identification of the headache site, the details of acupoint selection can be seen in the previously published protocol (J. Chen et al. 2022). Both TA and SA were replaced by the park sham device (PSD). In the TA group, deqi sensation (soreness, numbness, bloating, or radiation sensation) was required at each acupoint, while the tip of PSD in the SA group was blunt and would not penetrate the skin. None of the patients were taking any medication to prevent MwoA during the trial. Ibuprofen (Sino‐american Tianjin Shike Pharmaceutical Co., LTD), containing a 300‐mg extended‐release capsule, was permitted in patients presenting with intolerable headaches, and patients were asked to record ibuprofen use in a headache diary.

### 2.4 Outcome Measurements

This research focused on the change in ALFF, fALFF, ReHo, and DC as its primary endpoint. Clinical assessments included: (1) the frequency of migraine attacks, defined as the number of migraine headaches with a pain‐free interval of at least 48 h; (2) days of a migraine attack; (3) pain intensity, using VAS (0 ‐ 10 cm); and (4) duration of each migraine attack (hours). In addition, headache impact test‐6 (HIT‐6) scores, and migraine specific quality of life question (MSQ) scores were used to evaluate the quality of life of the participants. Participants were evaluated before treatment and after four weeks of treatment.

### 2.5 MRI Data Acquisition and Preprocessing

Sessions of fMRI scanning were performed in the MRI room of the hospital of Chengdu university of TCM. Patients should be free of migraine attacks for at least 72 h at the time of scanning, and brain images were acquired with a 3.0 Tesla system (GE discovery MR750, GE healthcare) with an eight‐channel head coil system. The resting‐state BOLD functional maps were acquired using single gradient echo‐planar imaging (EPI, repetition time = 2000 ms, echo time = 30 ms, data matrix 64 × 64, field of view 240 × 240 mm^2^, flip angle = 90 degrees, slice thickness = 5 mm, and voxel size = 3.75 × 3.75 × 5 mm^3^). The scan lasted for seven  min, and 205 time points were obtained. During the fMRI scan, all participants were asked not to move their heads, to close their eyes, to plug their ears, to remain conscious, and to avoid wandering thoughts.

### 2.6 MRI Data Preprocessing

The fMRI images were preprocessed using statistical parametric mapping eight (SPM8, http://www.fil.ion.ucl.ac.uk/spm), and data processing and analysis for brain imaging tools (C.‐G. Yan et al. 2016, http://rfmri.org/DPABI). The steps were as follows: (1) remove the first five time points; (2) slice time and head motion correction; (3) images of each participant were normalized to T1 using an EPI template; (4) T1 image were normalized to Montreal neurological institute (MNI) space; (5) spatial smoothing was performed using a 6 mm Gaussian kernel with full width at half maximum, and ReHo was calculated before smoothing; and (6) eliminating the linear trend of the data. Participants with head translations exceeding 2.0 mm or rotations exceeding 2.0° during the scan were excluded.

### 2.7 MRI Data Calculation

In this study, we extracted four rs‐MRI metrics, including ALFF, fALFF, ReHo, and DC. The location assignment of T1 maps was based on the automated anatomical labelling (AAL) atlas (Tzourio‐Mazoyer et al. 2002). After data preprocessing, fast Fourier transform was used to transform the BOLD signal time series to the frequency domain to obtain the power spectrum. The square root of the power spectrum of a given voxel in the range of 0.01 to 0.08 Hz (Y.‐F. Zang et al. 2007) was calculated and averaged, and the average square root value was the ALFF value. The fALFF value was calculated by dividing the power within the ALFF by the total power in the entire measurable frequency range (Zuo et al. 2010).

Subsequently, to calculate ReHo and DC, temporal filtering between 0.01 and 0.1 Hz was applied. ReHo was calculated by calculating the Kendall concordance coefficient of a given voxel and its 26 neighboring voxels in the same time series to generate a ReHo map for each subject (Y. Zang et al. 2004). In DC, Pearson correlation was used to correlate each voxel with all other voxels in the whole brain based on time series to obtain the correlation coefficient matrix. The threshold (*r* > 0.25, *p* ≤ 0.001) was used to eliminate the low temporal correlation caused by signal noise, and the DC value was obtained by calculating the sum of positive connection weights in a single voxel (Zuo et al. 2012).

### 2.8 Statistical Analysis

According to the neuroimaging sample size reported in the relevant literature, a relatively stable result could be obtained with 20 samples in each group(Szucs and Ioannidis 2020). Considering factors such as patient withdrawal during treatment and incomplete data in MRI scans, we set the inclusion of 32 patients in each group, so a total of 64 patients were required to be included in this study.

#### 2.8.1 Clinical Data Analysis

All clinical data were analyzed using SPSS 26.0. The normal distribution of the data was assessed using visual inspection of histograms and the Shapiro‐Wilk test. Data that did not follow a normal distribution were analyzed by nonparametric tests. For continuous variables, the median (lower quartiles; upper quartiles) was used for description, and dichotomous variables were described using frequencies and percentages (*N*, %). Statistical analysis was performed using a two‐tailed test with the significance level set at 5%. We used the chi‐square test, Mann–Whitney U test, and Wilcoxon's signed rank test to analyze the clinical data. An exploratory subgroup analysis of all clinical outcomes for the use of ibuprofen during treatment was performed using linear regression models.

#### 2.8.2 MRI Data Analysis

All fMRI parameters were normalized using Fisher's r‐to‐z score normalization procedure. The ALFF, fALFF, ReHo, and DC values were compared within and between the two treatment groups, respectively, using SPM8. Two‐sample t tests were used to compare post‐treatment values between the TA and SA groups, and paired t tests were used to compare pre‐treatment and post‐treatment values in each group. Individual age, sex, and head movement parameters (mean framewise displacement, Jenkinson) were used as covariates, and rs‐fMRI data were also treated as covariates for post‐treatment group comparisons. Voxel thresholds *p* < 0.001, and cluster levels *p* < 0.05 (Gaussian random field, GRF) were used for all analyses. A general linear model was used for subgroup analysis of ibuprofen use. Spearman correlation analysis (SPSS 26.0) was used to assess the correlation between ALFF, fALFF, ReHo, DC, and clinical indicators. Only associations with *p* < 0.05 were considered significant.

## 3 Results

**FIGURE 2:**
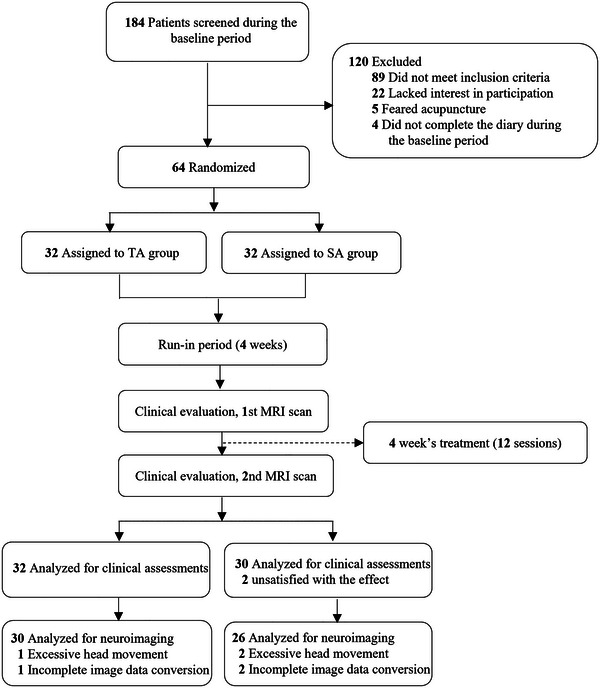
Study flow chart. Abbreviation: SA, sham acupuncture; TA, true acupuncture.

After 184 patients were screened, 64 patients with MwoA were enrolled in the study and randomly assigned to the TA and the SA groups. Two patients in the TA group and four patients in the SA group with excessive head movement and incomplete image data conversion, and two patients in the SA group dropped out due to unsatisfactory efficacy. Finally, 62 patients (32 in the TA group and 30 in the SA group) were included in the clinical analysis, and 56 patients (30 in the TA group and 26 in the SA group) were included in the imaging analysis (Figure 2).

### 3.1 Baseline Characteristics

The baseline demographic characteristics of all patients are summarized in Table 1. There was no significant variation between the two groups in gender, age, body mass index (BMI), duration of illness, education level, migraine attacks, proportion of using ibuprofen, and quality of life (HIT‐6 score and MSQ score) (*p* > 0.05).

**TABLE 1:** Baseline characteristics of the patients, values are M (P25, P75) unless stated otherwise.

Characteristics	TA (*N* = 32)	SA (*N* = 30)	*P*‐value
Age, years	33.00 (25.00, 43.00)	32.00 (26.75, 41.50)	0.735
No (%) women	22 (68.75%)	25 (83.33%)	0.240
BMI (kg/m^2^)	21.80 (19.65, 24.57)	20.68 (18.49, 23.18)	0.195
Education, months	13.50 (9.00, 16.00)	16.00 (14.75, 17.00)	0.069
Duration of illness, months	77.00 (36.00, 100.75)	88.50 (60.00, 144.00)	0.065
Frequency of migraine Attacks	4.50 (3.00, 5.00)	3.00 (3.00, 5.00)	0.471
Days with migraine	4.50 (3.00, 5.75)	3.00 (2.00, 5.00)	0.147
VAS score	5.20 (4.06, 6.58)	5.75 (4.93, 6.13)	0.707
Duration of each migraine attack	6.28 (3.05, 14.25)	7.90 (0.75, 12.00)	0.549
No (%) use of ibuprofen	20 (62.50%)	14 (46.67%)	0.307
HIT‐6 score	63.50 (58.00, 67.00)	65.00 (59.00, 67.00)	0.783
MSQ score, role restrictive domain	64.29 (49.29, 80.00)	62.86 (57.14, 65.71)	0.860
MSQ score, role preventive domain	80.00 (61.25, 88.75)	75.00 (63.75, 85.00)	0.655
MSQ score, emotional domain	80.00 (66.67, 93.33)	83.34 (66.67, 93.33)	0.949

Abbreviations: BMI, body mass index; HIT‐6, headache impact test‐6; MSQ, migraine specific quality of life questionnaire; SA, sham acupuncture; TA, true acupuncture; VAS, visual analog scale.

### 3.2 Medication Uses

During the four‐week treatment period of this study, nine (28.1%) patients in the TA group and 12 (40.0%) patients in the SA group reported the use of acute analgesics (ibuprofen), with no statistically significant difference in the number of patients between the two groups (*p* > 0.05; Table 2). The interval between the time of medication and the MRI scan was more than 24 h in all patients.

**TABLE 2:** Clinical outcome measures in each group, values are M (P25, P75) unless stated otherwise.

Outcome measure	TA (n = 32)	SA (n = 30)	*p^a^*
Frequency of migraine attacks			
End—baseline	−1.00 (‐2.75, ‐1.00)	0.00 (‐2.00, 1.00)	0.014
*p*b	< 0.01	0.417	
Days of migraine attacks			
End—baseline	−1.00 (‐3.00, 0.00)	0.00 (‐2.00, 1.00)	0.020
*p*b	< 0.01	0.645	
VAS score			
End—baseline	−1.95 (‐3.00, ‐0.35)	−0.25 (‐1.13, 0.14)	0.012
*p*b	< 0.01	0.016	
Duration of each migraine attack			
End—baseline	−2.40 (‐7.85, 0.00)	−0.13 (‐3.05, 0.19)	0.050
*p*b	<0.01	0.301	
Use of acute pain medication			
End of treatment (%)	9 (28.13)	12 (40.00)	0.423
HIT‐6 score			
End—baseline	−7.50 (‐16.50, 0.00)	−4.50 (‐9.25, 1.00)	0.135
*p*b	< 0.01	< 0.01	
MSQ score, role restrictive domain			
End—baseline	7.15 (‐4.99, 34.29)	10.00 (0.00, 20.00)	0.626
*p*b	< 0.01	0.012	
MSQ score, role preventive domain			
End—baseline	10.00 (‐3.75, 23.75)	5.00 (0.00, 20.00)	0.681
*p*b	< 0.01	0.028	
MSQ score, emotional functioning domain			
End—baseline	13.33 (‐6.67, 26.67)	6.66 (‐8.33, 13.33)	0.329
*p*b	0.037	0.257	

Abbreviations: HIT‐6, headache impact test‐6; MSQ, migraine specific quality of life questionnaire; SA: sham acupuncture; TA: true acupuncture; VAS, visual analog scale.

Annotation: *p*a, comparison between the two groups; *p*b, within‐group comparisons.

### 3.3 Clinical Outcomes

After four weeks of treatment, the changes in migraine frequency, migraine days, and VAS scores in the TA group were statistically different from those in the SA group (*p* < 0.05, Table 2). However, there were no statistical differences in the duration of each migraine attack, HIT‐6 score and MSQ score between the two groups (*p* > 0.05; Table 2).

Compared with the baseline, the TA group showed significantly improvement in the migraine frequency, migraine days, VAS scores, duration of each migraine attack, HIT‐6 scores, and MSQ scores after treatment (*p* < 0.05, Table 2); whereas only VAS, HIT‐6, and MSQ scores significantly improved in the SA group (*p* < 0.05, Table 2). In the exploratory subgroup analysis, the effects of acupuncture on all clinical measures were consistent, and the use of ibuprofen during treatment did not affect the treatment effect (all interaction *p* > 0.05; Supplementary Figure 1).

### 3.4 Neuroimaging Results

#### 3.4.1 Modulation Effects of True Acupuncture

**FIGURE 3:**
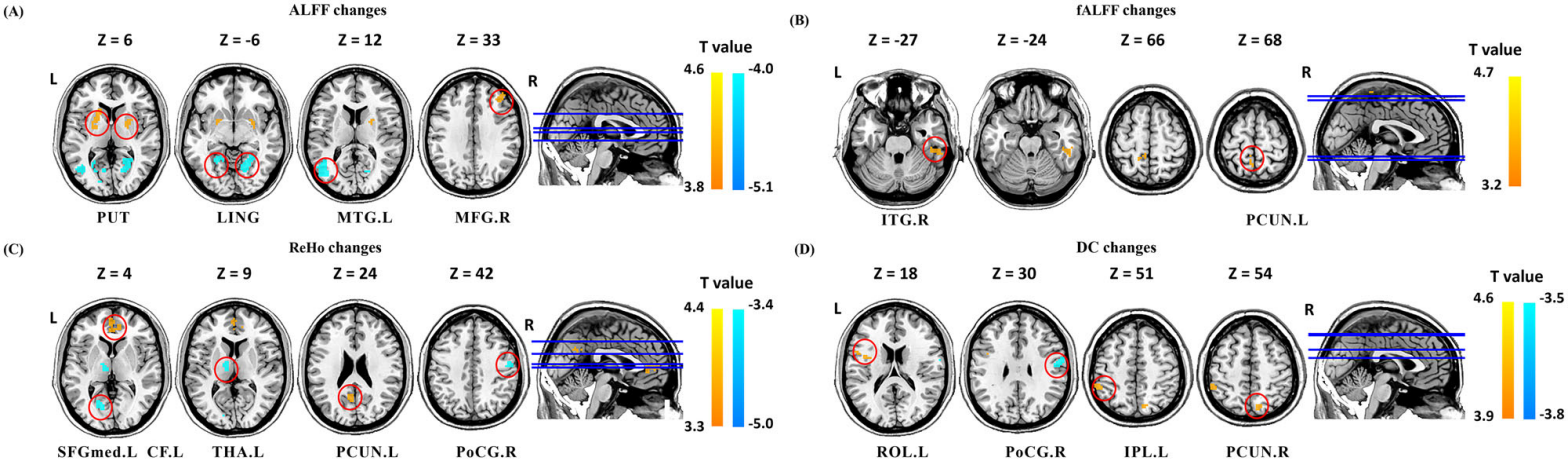
Resting state ALFF, fALFF, ReHo, and DC changes of MwoA patients in TA group (*p* < 0.05, GRF corrected). Abbreviation: ALFF, frequency fluctuation; CF, calcarine fissure; DC, degree centrality; fALFF, fractional amplitude of low frequency fluctuations; IPL, inferior parietal lobule; ITG, inferior temporal gyrus; L, left; LING: lingual gyrus; MFG, middle frontal gyrus; MTG, middle temporal gyrus; PCUN, precuneus; PoCG, postcentral gyrus; PUT, Putamen; R: right; ReHo, regional homogeneity; ROL, Rolandic operculum; SFGmed, superior frontal medial; TA, true acupuncture; THA, thalamus. Notes: (A) brain regions showed altered ALFF value after TA treatment; (B) brain regions showed altered fALFF value after TA treatment; (C) brain regions showed altered ReHo value after TA treatment; And (D) brain regions showed altered DC value after TA treatment.

After TA treatment, the ALFF values increased in the bilateral putamen and right middle frontal gyrus, and decreased in the bilateral lingual gyrus and left middle temporal gyrus. The fALFF values increased in the right inferior temporal gyrus and the left precuneus. The ReHo values increased in the left medial superior frontal gyrus and precuneus, and decreased in the left calcarine fissure, thalamus, and right postcentral gyrus. DC values increased in the left rolandic operculum, inferior parietal lobule, and right precuneus, and decreased in the right postcentral gyrus (Figure 3, Table 3).

**TABLE 3:** ALFF, fALFF, ReHo and DC changes in patients with MwoA after TA.

Brain region	Hemi	MNI coordinate			Voxels	Change	T value
		X	y	z			
ALFF							
Putamen	R	24	0	6	138	↑	4.56
Putamen	L	−21	12	6	97	↑	4.34
Lingual gyrus	R	21	−60	−6	201	↓	−3.92
Lingual gyrus	L	−27	−54	−3	100	↓	−4.11
Middle temporal gyrus	L	−42	−66	12	117	↓	−5.07
Middle frontal gyrus	R	36	36	33	63	↑	3.94
fALFF							
Inferior temporal gyrus	R	54	−33	−27	44	↑	4.63
Precuneus	L	−6	−51	66	43	↑	3.26
ReHo							
Medial superior frontal gyrus	L	0	54	6	74	↑	4.36
Calcarine fissure	L	−21	−69	3	44	↓	−4.91
Thalamus	L	−12	−9	9	55	↓	−4.72
Precuneus	L	−6	−60	24	40	↑	3.39
Precentral gyrus	R	54	−9	42	42	↓	−3.41
DC							
Rolandic operculum	L	−39	0	18	40	↑	3.56
Postcentral gyrus	R	57	−9	30	60	↓	−3.79
Inferior parietal lobule	L	−51	−42	51	40	↑	4.50
Precuneus	R	12	−72	54	46	↑	3.43

Abbreviations: ALFF, amplitude of low frequency fluctuations; DC: degree centrality; fALFF: fractional amplitude of low frequency fluctuations; Hemi, hemisphere; MNI, montreal neurological institute; MwoA, migraine without aura; ReHo, regional homogeneity; TA, true acupuncture.

Annotation: GRF corrected, *p* < 0.05; ↑, increase; ↓, decrease.

#### 3.4.2 Modulation Effects of Sham Acupuncture

**FIGURE 4:**
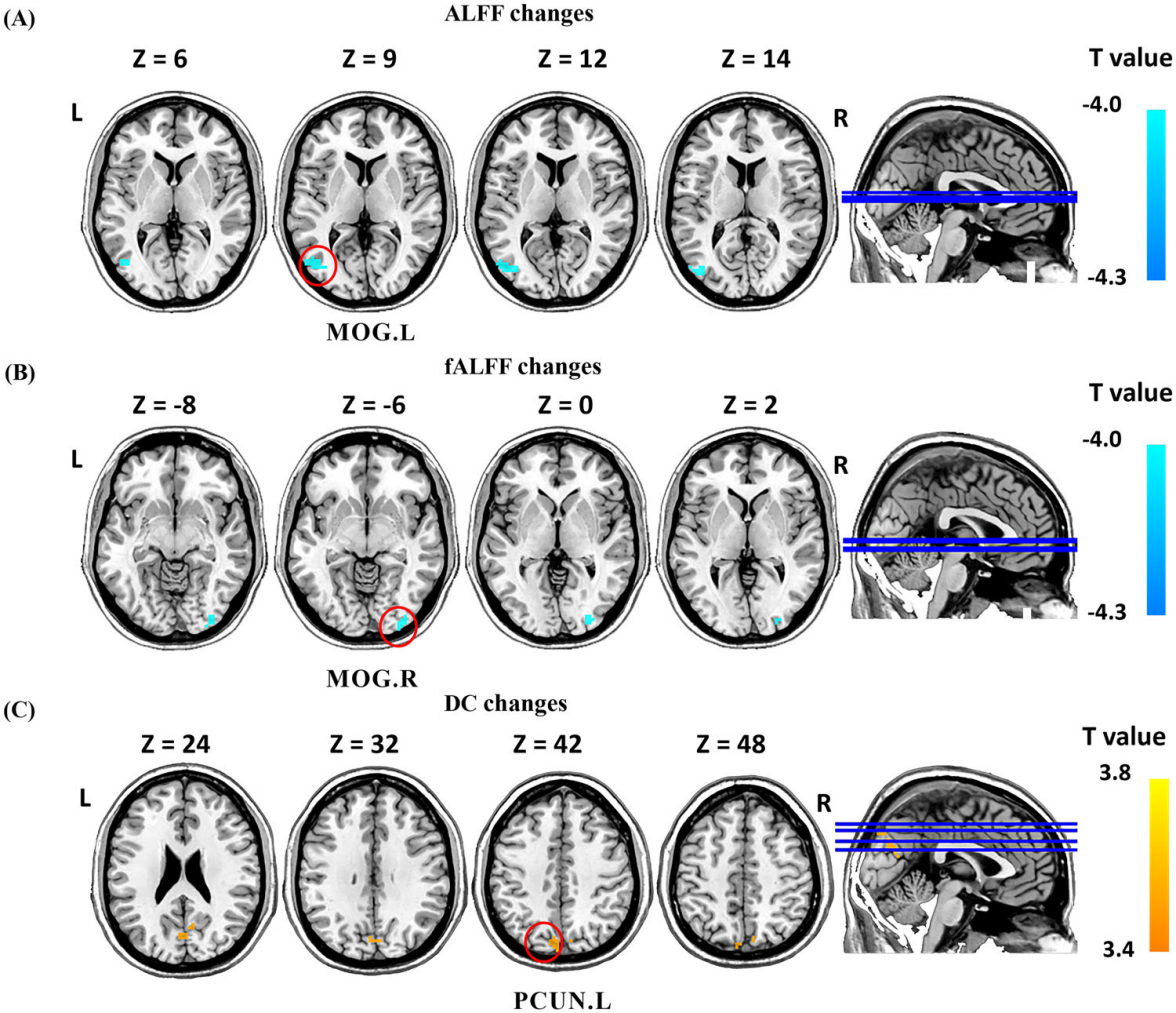
Resting state fALFF and DC changes of MwoA patients in SA group (*p* < 0.05, GRF corrected). Abbreviation: ALFF, frequency fluctuation; DC, degree centrality; fALFF, fractional amplitude of low frequency fluctuations; L, left; R, right; MOG, middle occipital gyrus; PCUN, precuneus; ReHo, regional homogeneity; SA, sham acupuncture. Notes: (A) brain regions showed altered fALFF value after SA treatment; and (B) brain regions showed altered DC value after SA treatment. Warm colors indicate increases, cool colors indicate decreases.

After SA treatment, DC values increased only in the left precuneus, and ALFF values in the left middle occipital gyrus and fALFF values in the right middle occipital gyrus decreased (Figure 4, Table 4).

**TABLE 4:** fALFF and DC changes in patients with MwoA after SA.

Brain region	Hemi	MNI coordinate			Voxels	Change	T value
		x	y	z			
ALFF							
Middle occipital gyrus	L	−48	−75	9	48	↓	−3.27
fALFF							
Middle occipital gyrus	R	−27	−90	0	46	↓	−3.46
DC							
Precuneus	L	−6	−78	42	85	↑	4.01

Abbreviations: DC: degree centrality; fALFF: fractional amplitude of low frequency fluctuations; Hemi, hemisphere; MNI, montreal neurological institute; MwoA, migraine without aura; SA, sham acupuncture.

Annotation: GRF corrected, *p* < 0.05; ↑, increase; ↓, decrease.

#### 3.4.3 Comparison of Modulation Effects in True Acupuncture and Sham Acupuncture

**FIGURE 5:**
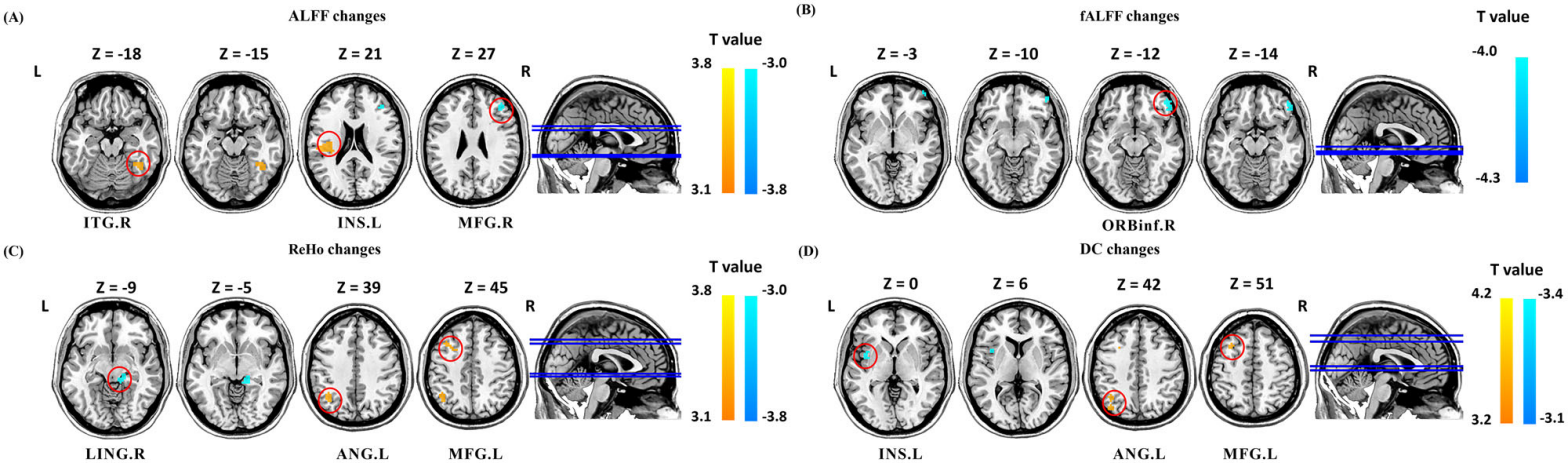
Comparison of ALFF, fALFF, ReHo and DC in resting state between the TA and SA in MwoA patients (*p* < 0.05, GRF corrected). Abbreviation: ALFF, frequency fluctuation; ANG, angular; DC: degree centrality; fALFF, fractional amplitude of low frequency fluctuations; INS, insula; L, left; ITG, inferior temporal gyrus; LING, lingual gyrus; MFG, middle frontal gyrus; ORBinf, inferior frontal orbit; R, right; ReHo, regional homogeneity; TA, true acupuncture. Notes: (A) brain regions showed altered ALFF value in the TA group compared to the SA group; (B) brain regions showed altered fALFF value in the TA group compared to the SA group; (C) brain regions showed altered ReHo value in the TA group compared to the SA group; (D) brain regions showed altered DC value in the TA group compared to the SA group. Warm colors indicate increases, cool colors indicate decreases.

After four weeks of treatment, compared with the SA group, the ALFF values increased in the right inferior temporal gyrus and left insula, and decreased in the right middle frontal gyrus in the TA group. The fALFF values decreased in the right inferior orbitofrontal gyrus, the ReHo values increased in the left angular gyrus and middle frontal gyrus; and decreased in the right lingual gyrus, the DC values increased in the left angular gyrus and middle frontal gyrus and decreased in the left insula (Figure 5, Table 5). Subgroup analysis showed an interaction between ibuprofen use and group in ALFF values in the right lingual gyrus and left suboccipital gyrus (Supplementary Figure 2; Supplementary Table 1), suggesting that ibuprofen use might affect neural activity in these brain regions by TA and SA.

**TABLE 5:** ALFF, fALFF, ReHo, and DC changes in patients with MwoA after TA and SA.

Brain region	Hemi	MNI coordinate			Voxels	Change	T value
		x	y	z			
ALFF							
Inferior temporal gyrus	R	45	−51	−18	48	↑	3.20
Middle frontal gyrus	R	42	36	27	64	↓	−3.35
Insula	L	−33	−24	21	75	↑	3.44
fALFF							
Inferior frontal orbit	R	51	45	−12	57	↓	−4.19
ReHo							
Lingual	R	15	−30	−9	39	↓	−4.29
Angular	L	−36	−60	39	66	↑	4.42
Middle frontal gyrus	L	−36	21	45	71	↑	4.42
DC							
Insula	L	−39	3	0	41	↓	−3.40
Angular	L	−39	−57	42	42	↑	3.88
Middle frontal gyrus	L	−30	18	51	35	↑	3.97

Abbreviations: ALFF, amplitude of low frequency fluctuations; DC: degree centrality; fALFF: fractional amplitude of low frequency fluctuations; Hemi, hemisphere; MNI, montreal neurological institute; MwoA, migraine without aura; ReHo, regional homogeneity; SA, sham acupuncture; TA, true acupuncture.

Annotation: GRF corrected, *p* < 0.05; ↑, increase; ↓, decrease.

#### 3.4.4 Correlation With the Clinical Improvements

**FIGURE 6:**
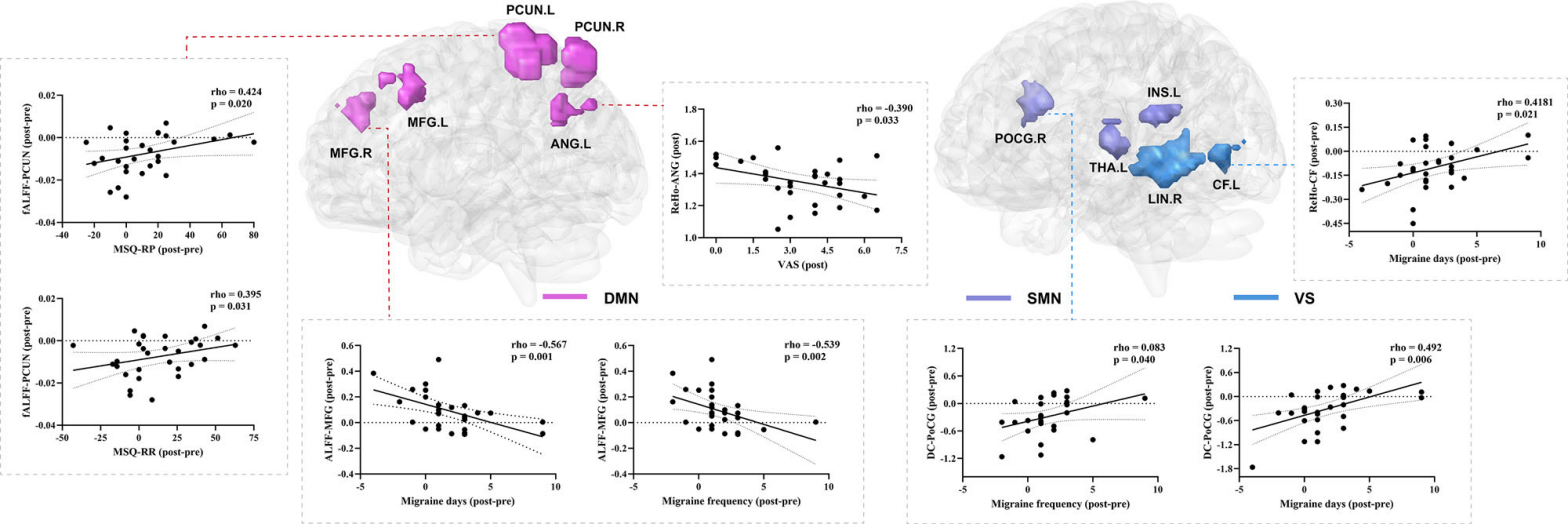
Correlation analysis between brain regions and clinical improvement of patients. Abbreviation: ALFF, frequency fluctuation; ANG, angular; CF, calcarine fissure; DC, degree centrality; DMN, default mode network; fALFF, fractional amplitude of low frequency fluctuations; L, left; MFG, middle frontal gyrus; MSQ‐RR, migraine specific quality of life questionnaire, role restrictive domain; MSQ‐RP, migraine specific quality of life questionnaire, role preventive domain; PCUN: precuneus; PoCG: postcentral gyrus; ReHo: regional homogeneity; R: right; SA: sham acupuncture; SMN: sensorimotor network; TA: true acupuncture; VAS: visual analog scale; VN: visual network.

Correlation analysis showed that after longitudinal treatment, the change in ALFF in the right middle frontal gyrus in the TA group was inversely correlated with the reduction in migraine frequency and migraine days. The change in ReHo in the left calcarine fissure was positively correlated with the reduction in migraine days, and the change in DC in the right postcentral gyrus was positively correlated with the reduction in migraine days and migraine frequency. The change in fALFF in the left precuneus is positively correlated with the improvement in quality of life (Figure 6). However, no correlation was found in the SA group. Compared with the SA group, the change in ReHo in the left angular gyrus was inversely correlated with the decrease in VAS in the TA group (Figure 6).

### 3.5 Patient Safety

All 64 patients were monitored for safety. During the four‐week treatment period, two patients (both in the TA group) reported adverse events. One patient had a subcutaneous hemorrhage in the needle insertion area, and one patient had local pain in the needle insertion area after acupuncture. All adverse events were reported as mild or moderate and did not require specific medical intervention. After one week of follow‐up, the patient fully recovered from the adverse event and did not withdraw from the trial.

## 4 Discussion

In this study, we investigated the effects of TA and SA on clinical improvement in MwoA patients and their modulation at voxel, local, and global scales. Compared with the SA group, the TA group showed superior benefits in clinical improvement of MwoA patients, and voxel in different space scales of brain function modulation degree and range of influence is more significant. TA could modulate the DMN (precuneus, middle frontal gyrus, superior frontal gyrus, inferior parietal lobule, angular gyrus), VN (lingual gyrus, calcarine fissure, middle temporal gyrus, inferior temporal gyrus), and SMN (postcentral gyrus, thalamus, insula) at different spatial voxel scales to improve the attack of migraine, reduce the intensity of pain, and improve the quality of life.

In this study, we found that after four weeks of treatment, both TA and SA improved the symptoms of MwoA patients, and TA was significantly superior to SA in improving the frequency of migraine attacks, migraine days, and pain intensity in patients. This finding is similar to the results of our previous RCT study of the efficacy of acupuncture for migraine prophylaxis (Zhao et al. 2017), which demonstrates the analgesic effects of acupuncture at acupoints in painful disorders (Qiao et al. 2020). The therapeutic effect of acupuncture on migraine, for a long time in the past, was considered to be a magical placebo effect (Gelfand 2017; McGeeney 2015). We hypothesized that the improvement in these clinical symptoms in the SA group might be related to placebo effects and psychological effects. We chose a control intervention with no needling at acupoints, and although the skin was not pierced, patients felt pain, which may have given the patient the illusion of needling. Previous studies have shown that the depth of needle insertion and the location of puncture directly influence the placebo effect (Kong et al. 2013). Admittedly, although the no‐insertion approach could have largely avoided the placebo effect, it still played a role.

After longitudinal treatment, ALFF, fALFF, ReHo, and DC values were changed in several brain regions of the DMN (precuneus, middle frontal gyrus, superior frontal gyrus, and inferior parietal lobule) in the TA group, and DC values in the precuneus also increased in the SA group. In the TA group, the change of fALFF value in precuneus was positively correlated with the increase in MSQ value, and the change in ALFF value in the middle frontal gyrus was inversely correlated with the reduction in migraine frequency and migraine days. The ALFF, ReHo, and DC values of the DMN (middle frontal gyrus and angular gyrus) were changed in the TA group compared with the SA group. The change in ReHo value in the angular gyrus was inversely correlated with lower VAS scores. The results based on multi‐spatial voxel scales suggest that the DMN, with precuneus as a key node, might be a key brain region for acupuncture efficacy.

The precuneus belongs to the parietal cortical structure and, as a major component of the DMN, is associated with pain sensitization, aberrant pain processing, and emotion processing (Goffaux et al. 2014). The middle frontal gyrus and superior frontal gyrus are part of the prefrontal cortex, which is sensitive to painful stimulation (Qi et al. 2022), influences the descending pain pathway (Lorenz et al. 2003) and is a component of the default network pattern. The DMN is a key node in pain modulation, and previous studies have found that migraine patients often have abnormalities in multiple brain regions of the DMN (C. Li et al. 2023). It has been found that in patients with pain and chronic pain, the ALFF values are reduced in the precuneus, middle frontal gyrus, superior frontal gyrus, and angular gyrus (Yu et al. 2022; Pan et al. 2018; Kang et al. 2023; Huang et al. 2020); the ReHo values are reduced in the precuneus (C. Chen et al. 2019), the DC values of the angular gyrus are decreased (Y.‐C. Yang et al. 2024); and the functional connectivity between the precuneus, the angular gyrus and other brain regions is also decreased (Ke et al. 2020; Zhe et al. 2023). Previous studies have shown that acupuncture can increase the ALFF values of the precuneus, superior frontal gyrus, and middle frontal gyrus (Yu et al. 2022; Pan et al. 2018; Kang et al. 2023), and the ALFF(Kang et al. 2023) and ReHo values of the angular gyrus in patients with pain(Kang et al. 2023; Liu et al. 2021), which are similar to the findings in our study. Correlation analysis suggested that TA can improve migraine and quality of life by modulating spontaneous neural activity, regional coherence, and functional importance of the DMN (precuneus, middle frontal gyrus, and angular gyrus).

After longitudinal treatment, the ReHo and DC values of the SMN (postcentral gyrus, thalamus) decreased in the TA group. Changes in DC values in the postcentral gyrus were positivelyassociated with reductions in migraine frequency and days. The ALFF and DC values of the SMN (insula) were altered in the TA group compared with the SA group. The results suggest that TA might play a therapeutic role by modulating the SMN. The postcentral gyrus is a key link in the SMN of pain processing (Frot et al. 2012), and it works together with the thalamus and insula to realize the comprehensive processing of pain (Messina et al. 2022), (You et al. 2022). It has been found that ALFF value (J. Yang et al. 2019), ReHo value (Wen et al. 2023), DC value (C.‐Q. Yan et al. 2020) and functional connectivity (Ke et al. 2020), in the postcentral gyrus are increased to varying degrees in patients with migraine and chronic pain. The ReHo values of the insula (C. Chen et al. 2019) and DC values of the posterior insula (Ke et al. 2020) were increased, and the synchronization or coordination of local thalamic functional activities was abnormal (Thiebaut de Schotten et al. 2014; Y.‐C. Yang et al. 2024; Yu et al. 2022). Acupuncture and transcutaneous auricular vagus nerve therapy reduce DC values and functional connectivity in the postcentral gyrus in patients with chronic pain (C.‐Q. Yan et al. 2020)^,^(Rao et al. 2023), which is similar to our findings. Correlation analysis showed that TA ameliorated migraine by reducing the functional importance of the SMN (postcentral gyrus).

After longitudinal treatment, the ALFF, fALFF, and ReHo of the VN (lingual gyrus, middle temporal gyrus, inferior temporal gyrus, calcarine fissure) in the TA group changed. The change in ReHo of the calcarine fissure was positively correlated with the decrease in migraine days. ALFF and ReHo values of the VN (inferior temporal gyrus, lingual gyrus) were altered in the TA group compared with the SA group. This suggests that the VN might be an effective brain network for TA at different spatial voxel scales. The calcarine fissure is involved in the integration of visual information (Thiebaut de Schotten et al. 2014), the lingual gyrus is an important part of the occipital visual area; the middle temporal and inferior temporal gyri are involved in visual network processing (Cao et al. 2022; Kemik et al. 2023). Strong evidence suggests that partial visual processing networks are involved in pain perception and modulation of chronic migraine (Puledda et al. 2019; Yuan et al. 2022). Visual processing dysfunction in the lingual gyrus is associated with light perception in migraine patients(Schankin et al. 2014), which may explain symptoms such as photophobia and visual aura in patients. Previous studies have also confirmed that the ALFF value, ReHo value, and functional connectivity with other brain regions of the calcarine fissure, lingual gyrus, and temporal cortex are abnormal in patients with pain(Bu et al. 2023; J. Yang et al. 2019; Yuan et al. 2022; Zhu et al. 2024). We found that TA could modulate abnormal neural activity in the VN at different spatial voxel scales. Correlation analysis suggested that TA could reduce migraine days by regulating the regional coherence of the VN (calcarine fissure).

 In addition, in the subgroup analysis of medication use, we found no interaction between ibuprofen and intervention in clinical outcomes, while neuroimaging results found interactive brain regions. It might be that alterations in brain functional activity do not necessarily translate directly into alterations in behavior or clinical symptoms. Furthermore, the results of the subgroup analyses are exploratory, and further studies are needed to verify in the future.

## 5 Limitations

This study has some limitations. First, we did not compare MwoA patients with healthy participants, which may have prevented us from exploring the pathogenesis of MwoA in detail. Second, patients who did not undergo neuroimaging scans during the follow‐up period, could not determine the potential neuroimaging mechanism in acupuncture in migraine prevention. Third, this was a single‐center trial, which may lead to unavoidable selection bias and analytical bias, affecting the accuracy of the results. Future multicenter, large‐sample, long‐term follow‐up studies are needed to support our results.

## 6 Conclusion

In conclusion, the therapeutic effect of TA on MwoA was superior to that of SA. TA can significantly reduce the frequency, days, and pain intensity of migraine in MwoA patients, and other clinical symptoms also have a tendency to improve. TA could modulate the spontaneous neural activity, regional coherence, and functional importance of the DMN, VN, and SMN at different spatial voxel scales, which may be the neuroimaging mechanism of acupuncture for MwoA.

## Author Contributions

**Chaorong Xie**: writing–original draft; visualization; methodology; software; conceptualization. **Zhiyang Zhang**: Software; data curation; validation. **Yutong Zhang**: Data curation; validation. **Xixiu Ni**: Formal analysis; methodology. **Yang Yu**: Formal analysis; visualization. **Xiaoyu Gao**: Conceptualization; investigation. **Mingsheng Sun**: Investigation; resources. **Xiao Wang**: Writing–review and editing; funding acquisition; methodology. **Ling Zhao**: Writing–review and editing; funding acquisition; project administration; supervision.

## Conflicts of Interest

The authors declare no conflicts of interest.

### Peer Review

The peer review history for this article is available at https://publons.com/publon/10.1002/brb3.70536

## Supporting information



Supplementary FIGURE 1. Subgroup analysis of clinical outcome measures in patients with ibuprofen use.
Supplementary FIGURE 2. Subgroup analyses of resting state for group and ibuprofen use interactions (*p* interaction < 0.05, GRF corrected). Supplementary TABLE 1. Subgroup analyses of resting state for group and ibuprofen use interactions.

## Data Availability

The datasets used and/or analyzed during the current study are available from the corresponding author on reasonable request.

## References

[brb370536-bib-0001] Ashina, M. 2020. “Migraine.” The New England Journal of Medicine 383, no. 19: 1866–1876. 10.1056/NEJMra1915327. 33211930

[brb370536-bib-0002] Ashina, M. , Z. Katsarava , T. P. Do , et al. 2021. “Migraine: Epidemiology and Systems of Care.” *The* Lancet 397, no. 10283: 10283. 10.1016/S0140-6736(20)32160-7. 33773613

[brb370536-bib-0003] Bu, C. , H. Ren , Q. Lv , et al. 2023. “Alteration of Static and Dynamic Intrinsic Brain Activity Induced by Short‐Term Spinal Cord Stimulation in Postherpetic Neuralgia Patients.” Frontiers in Neuroscience 17: 1254514. 10.3389/fnins.2023.1254514. 37877014 PMC10590878

[brb370536-bib-0004] Cao, Y. , H. Xie , H. Sun , et al. 2022. “Common and Distinct Patterns of Gray Matter Alterations in Young Adults With Borderline Personality Disorder and Major Depressive Disorder.” European Archives of Psychiatry and Clinical Neuroscience 272, no. 8: 1569–1582. 10.1007/s00406-022-01405-9. 35419633

[brb370536-bib-0005] Chen, C. , M. Yan , Y. Yu , et al. 2019. “Alterations in Regional Homogeneity Assessed by fMRI in Patients With Migraine Without Aura.” Journal of Medical Systems 43, no. 9: 298. 10.1007/s10916-019-1425-z. 31352647

[brb370536-bib-0006] Chen, J. , S. Zhou , M. Sun , et al. 2022. “Manual Acupuncture as Prophylaxis for Migraine Without Aura: Study Protocol for a Multi‐Center, Randomized, Single‐Blinded Trial.” Trials 23, no. 1: 574. 10.1186/s13063-022-06510-7. 35854329 PMC9295267

[brb370536-bib-0007] Chen, P.‐K. , and S.‐J. Wang . 2019. “Medication Overuse and Medication Overuse Headache: Risk Factors, Comorbidities, Associated Burdens and Nonpharmacologic and Pharmacologic Treatment Approaches.” Current Pain and Headache Reports 23, no. 8: 60. 10.1007/s11916-019-0796-7. 31346781

[brb370536-bib-0008] Chong, C. D. , T. J. Schwedt , and A. Hougaard . 2019. “Brain Functional Connectivity in Headache Disorders: A Narrative Review of MRI Investigations.” Journal of Cerebral Blood Flow and Metabolism: Official Journal of the International Society of Cerebral Blood Flow and Metabolism 39, no. 4: 650–669. 10.1177/0271678X17740794. 29154684 PMC6446420

[brb370536-bib-0009] Ferrari, M. D. , P. J. Goadsby , R. Burstein , et al. 2022. “Migraine.” Nature Reviews. Disease Primers 8, no. 1: 2655–2668. 10.1038/s41572-021-00328-4. 35027572

[brb370536-bib-0010] Frot, M. , M. Magnin , F. Mauguière , and L. Garcia‐Larrea . 2012. “Cortical Representation of Pain in Primary Sensory‐Motor Areas (S1/M1)—A Study Using Intracortical Recordings in Humans.” Human Brain Mapping 34, no. 10: 2367–2746. 10.1002/hbm.22097. 22706963 PMC6869910

[brb370536-bib-0011] Gelfand, A. A. 2017. “Acupuncture for Migraine Prevention: Still Reaching for Convincing Evidence.” JAMA Internal Medicine 177, no. 4: 516–517. 10.1001/jamainternmed.2016.9404. 28241222

[brb370536-bib-0012] Goffaux, P. , L. Girard‐Tremblay , S. Marchand , K. Daigle , and K. Whittingstall . 2014. “Individual Differences in Pain Sensitivity Vary as a Function of Precuneus Reactivity.” Brain Topography 27, no. 3: 366–374. 10.1007/s10548-013-0291-0. 23636269

[brb370536-bib-0013] Headache Classification Committee of the International Headache Society (IHS) The International Classification of Headache Disorders, 3rd edition . 2018. Cephalalgia: An International Journal of Headache 38, no. 1: 1–211. 10.1177/0333102417738202. 29368949

[brb370536-bib-0014] Huang, J. , Y. Li , H. Xie , et al. 2020. “Abnormal Intrinsic Brain Activity and Neuroimaging‐Based fMRI Classification in Patients With Herpes Zoster and Postherpetic Neuralgia.” Frontiers in Neurology 11: 532110. 10.3389/fneur.2020.532110. 33192967 PMC7642867

[brb370536-bib-0015] Kang, B. , C. Zhao , J. Ma , et al. 2023. “Electroacupuncture Alleviates Pain After Total Knee Arthroplasty Through Regulating Neuroplasticity: A Resting‐State Functional Magnetic Resonance Imaging Study.” Brain and Behavior 13, no. 3: e2913. 10.1002/brb3.2913. 36749304 PMC10013951

[brb370536-bib-0016] Ke, J. , Y. Yu , X. Zhang , et al. 2020. “Functional Alterations in the Posterior Insula and Cerebellum in Migraine without Aura: A Resting‐State MRI Study.” Frontiers in Behavioral Neuroscience 14: 567588. 10.3389/fnbeh.2020.567588. 33132860 PMC7573354

[brb370536-bib-0017] Kemik, K. , E. Ada , B. Çavuşoğlu , C. Aykaç , D. D. Emek‐Savaş , and G. Yener . 2023. “Functional Magnetic Resonance Imaging Study During Resting State and Visual Oddball Task in Mild Cognitive Impairment.” CNS Neuroscience and Therapeutics 30, no. 2: e14371. 10.1111/cns.14371. 37475197 PMC10848090

[brb370536-bib-0018] Kong, J. , R. Spaeth , A. Cook , et al. 2013. “Are all Placebo Effects Equal? Placebo Pills, Sham Acupuncture, Cue Conditioning and Their Association.” PLoS ONE 8, no. 7: e67485. 10.1371/journal.pone.0067485. 23935833 PMC3729687

[brb370536-bib-0021] Li, Y. , H. Zheng , C. M. Witt , et al. 2012. “Acupuncture for Migraine Prophylaxis: A Randomized Controlled Trial.” CMAJ: Canadian Medical Association 184, no. 4: 401–410. 10.1503/cmaj.110551. PMC3291669 22231691

[brb370536-bib-0022] Li, Z. , F. Zeng , T. Yin , et al. 2017. “Acupuncture Modulates the Abnormal Brainstem Activity in Migraine Without Aura Patients.” NeuroImage: Clinical 15: 367–375. 10.1016/j.nicl.2017.05.013. 28580293 PMC5447510

[brb370536-bib-0065] Li, C. , X. Li , K. He , et al. 2023. “Discovery of the Mechanisms of Acupuncture in the Treatment of Migraine Based on Functional Magnetic Resonance Imaging and Omics.” Frontiers of Medicine 17, no. 5: 993–1005. 10.1007/s11684-023-0989-7. 37389804

[brb370536-bib-0023] Linde, K. , G. Allais , B. Brinkhaus , et al. 2016. “Acupuncture for the Prevention of Episodic Migraine.” The Cochrane Database of Systematic Reviews 2016, no. 6: CD001218. 10.1002/14651858.CD001218.pub3. 27351677 PMC4977344

[brb370536-bib-0024] Liu, S. , S. Luo , T. Yan , et al. 2021. “Differential Modulating Effect of Acupuncture in Patients With Migraine Without Aura: A Resting Functional Magnetic Resonance Study.” Frontiers in Neurology 12: 680896. 10.3389/fneur.2021.680896. 34122321 PMC8193984

[brb370536-bib-0025] Lorenz, J. , S. Minoshima , and K. L. Casey . 2003. “Keeping Pain Out of Mind: The Role of the Dorsolateral Prefrontal Cortex in Pain Modulation.” Brain: A Journal of Neurology 126, no. 5: 1079–1091. 10.1093/brain/awg102. 12690048

[brb370536-bib-0026] Ma, P. , X. Dong , Y. Qu , et al. 2021. “A Narrative Review of Neuroimaging Studies in Acupuncture for Migraine.” Pain Research and Management 9460695. 10.1155/2021/9460695. 34804268 PMC8598357

[brb370536-bib-0027] McGeeney, B. E. 2015. “Acupuncture Is All Placebo and Here Is Why.” Headache 55, no. 3: 465–469. 10.1111/head.12524. 25660556

[brb370536-bib-0028] Messina, R. , C. Gollion , R. H. Christensen , and F. M. Amin . 2022. “Functional MRI in Migraine.” Current Opinion in Neurology 35, no. 3: 328–335. 10.1097/WCO.0000000000001060. 35674076

[brb370536-bib-0029] Messina, R. , M. A. Rocca , P. J. Goadsby , and M. Filippi . 2023. “Insights Into Migraine Attacks From Neuroimaging.” The Lancet Neurology 22, no. 9: 834–846. 10.1016/S1474-4422(23)00152-7. 37478888

[brb370536-bib-0030] Pan, Z.‐M. , H.‐J. Li , J. Bao , et al. 2018. “Altered Intrinsic Brain Activities in Patients With Acute Eye Pain Using Amplitude of Low‐Frequency Fluctuation: A Resting‐State fMRI Study.” Neuropsychiatric Disease and Treatment 14: 251–257. 10.2147/NDT.S150051. 29386898 PMC5767092

[brb370536-bib-0031] Puledda, F. , D. Ffytche , O. O'Daly , and P. J. Goadsby . 2019. “Imaging the Visual Network in the Migraine Spectrum.” Frontiers in Neurology 10: 1325. 10.3389/fneur.2019.01325. 31920945 PMC6923266

[brb370536-bib-0032] Qi, X. , K. Cui , Y. Zhang , et al. 2022. “A Nociceptive Neuronal Ensemble in the Dorsomedial Prefrontal Cortex Underlies Pain Chronicity.” Cell Reports 41, no. 11: 111833. 10.1016/j.celrep.2022.111833. 36516746

[brb370536-bib-0066] Qiao, L. , M. Guo , J. Qian , B. Xu , C. Gu , and Y. Yang . 2020. “Research Advances on Acupuncture Analgesia.” The American Journal of Chinese Medicine 48, no. 2: 245–258. 10.1142/S0192415X20500135. 32138535

[brb370536-bib-0035] Raichle, M. E. 2015. “The Brain's Default Mode Network.” Annual Review of Neuroscience 38: 433–447. 10.1146/annurev-neuro-071013-014030. 25938726

[brb370536-bib-0036] Rao, Y. , W. Liu , Y. Zhu , et al. 2023. “Altered Functional Brain Network Patterns in Patients With Migraine Without Aura After Transcutaneous Auricular Vagus Nerve Stimulation.” Scientific Reports 13: 9604. 10.1038/s41598-023-36437-1. 37311825 PMC10264378

[brb370536-bib-0037] Schankin, C. J. , F. H. Maniyar , T. Sprenger , D. E. Chou , M. Eller , and P. J. Goadsby . 2014. “The Relation Between Migraine, Typical Migraine Aura and “Visual Snow”.” Headache 54, no. 6: 957–966. 10.1111/head.12378. 24816400

[brb370536-bib-0038] Schramm, S. , C. Börner , M. Reichert , et al. 2023. “Functional Magnetic Resonance Imaging in Migraine: A Systematic Review.” Cephalalgia: An International Journal of Headache 43, no. 2: 3331024221128278. 10.1177/03331024221128278. 36751858

[brb370536-bib-0039] Stovner, L. J. , K. Hagen , M. Linde , and T. J. Steiner . 2022. “The Global Prevalence of Headache: an Update, With Analysis of the Influences of Methodological Factors on Prevalence Estimates.” The Journal of Headache and Pain 23, no. 1: 34. 10.1186/s10194-022-01402-2. 35410119 PMC9004186

[brb370536-bib-0040] Szucs, D. , and J. P. Ioannidis . 2020. “Sample Size Evolution in Neuroimaging Research: An Evaluation of Highly‐Cited Studies (1990‐2012) and of Latest Practices (2017‐2018) in High‐Impact Journals.” Neuroimage 221: 117164. 10.1016/j.neuroimage.2020.117164. 32679253

[brb370536-bib-0041] Terwindt, G. M. , M. D. Ferrari , M. Tijhuis , S. M. Groenen , H. S. Picavet , and L. J. Launer . 2000. “The Impact of Migraine on Quality of Life in the General Population: The GEM Study.” Neurology 55, no. 5: 624–629. 10.1212/wnl.55.5.624. 10980723

[brb370536-bib-0042] Thiebaut de Schotten, M. , M. Urbanski , R. Valabregue , D. J. Bayle , and E. Volle . 2014. “Subdivision of the Occipital Lobes: An Anatomical and Functional MRI Connectivity Study.” Cortex 56: 121–137. 10.1016/j.cortex.2012.12.007. 23312799

[brb370536-bib-0043] Tzourio‐Mazoyer, N. , B. Landeau , D. Papathanassiou , et al. 2002. “Automated Anatomical Labeling of Activations in SPM Using a Macroscopic Anatomical Parcellation of the MNI MRI Single‐Subject Brain.” Neuroimage 15, no. 1: 273–289. 10.1006/nimg.2001.0978. 11771995

[brb370536-bib-0044] Wen, J.‐J. , Y.‐Y. Gao , M.‐T. Li , et al. 2023. “Regional Abnormalities of Spontaneous Brain Activity in Migraine: A Coordinate‐based Meta‐Analysis.” Journal of Neuroscience Research 101, no. 8: 1205–1223. 10.1002/jnr.25191. 37001980

[brb370536-bib-0045] Xu, S. , L. Yu , X. Luo , et al. 2020. “Manual Acupuncture Versus Sham Acupuncture and Usual Care for Prophylaxis of Episodic Migraine Without Aura: Multicentre, Randomised Clinical Trial.” BMJ 368: m697. 10.1136/bmj.m697. 32213509 PMC7249245

[brb370536-bib-0046] Yan, C.‐G. , X.‐D. Wang , X.‐N. Zuo , and Y.‐F. Zang . 2016. “DPABI: Data Processing and Analysis for (Resting‐State) Brain Imaging.” Neuroinformatics 14, no. 3: 339–351. 10.1007/s12021-016-9299-4. 27075850

[brb370536-bib-0047] Yan, C.‐Q. , J.‐W. Huo , X. Wang , et al. 2020. “Different Degree Centrality Changes in the Brain After Acupuncture on Contralateral or Ipsilateral Acupoint in Patients With Chronic Shoulder Pain: A Resting‐State fMRI Study.” Neural Plasticity 5701042. 10.1155/2020/5701042. 32377180 PMC7197008

[brb370536-bib-0067] Yang, J. , B. Li , Q.‐Y. Yu , et al. 2019. “Altered Intrinsic Brain Activity in Patients With Toothaches Using the Amplitude of Low‐Frequency Fluctuations: A Resting‐State fMRI Study.” Neuropsychiatric Disease and Treatment 15: 283–291. 10.2147/NDT.S189962. 30697053 PMC6342150

[brb370536-bib-0050] Yang, Y.‐C. , X.‐Y. Wei , Y.‐Y. Zhang , et al. 2024. “Modulation of Temporal and Occipital Cortex by Acupuncture in Non‐Menstrual MWoA Patients: A Rest BOLD fMRI Study.” BMC Complementary Medicine and Therapies 24, no. 1: 43. 10.1186/s12906-024-04349-w. 38245739 PMC10799457

[brb370536-bib-0051] You, H.‐J. , J. Lei , and A. Pertovaara . 2022. “Thalamus: the ‘Promoter’ of Endogenous Modulation of Pain and Potential Therapeutic Target in Pathological Pain.” Neuroscience and Biobehavioral Reviews 139: 104745. 10.1016/j.neubiorev.2022.104745. 35716873

[brb370536-bib-0052] Yu, Z. , R.‐R. Wang , W. Wei , et al. 2022. “A Coordinate‐Based Meta‐Analysis of Acupuncture for Chronic Pain: Evidence From fMRI Studies.” Frontiers in Neuroscience 16: 1049887. 10.3389/fnins.2022.1049887. 36590302 PMC9795831

[brb370536-bib-0068] Yuan, Z. , W. Wang , X. Zhang , et al. 2022. “Altered Functional Connectivity of the Right Caudate Nucleus in Chronic Migraine: A Resting‐State fMRI Study.” The Journal of Headache and Pain 23, no. 1: 154. 10.1186/s10194-022-01506-9. 36460958 PMC9717534

[brb370536-bib-0055] Zang, Y. , T. Jiang , Y. Lu , Y. He , and L. Tian . 2004. “Regional Homogeneity Approach to fMRI Data Analysis.” Neuroimage 22, no. 1: 394–400. 10.1016/j.neuroimage.2003.12.030. 15110032

[brb370536-bib-0056] Zang, Y.‐F. , Y. He , C.‐Z. Zhu , et al. 2007. “Altered Baseline Brain Activity in Children With ADHD Revealed by Resting‐State Functional MRI.” Brain & Development 29, no. 2: 83–91. 10.1016/j.braindev.2006.07.002. 16919409

[brb370536-bib-0057] Zhang, H. , D. Shen , and W. Lin . 2019. “Resting‐State Functional MRI Studies on Infant Brains: A Decade of Gap‐Filling Efforts.” Neuroimage 185: 664–684. 10.1016/j.neuroimage.2018.07.004. 29990581 PMC6289773

[brb370536-bib-0058] Zhao, L. , J. Chen , Y. Li , et al. 2017. “The Long‐Term Effect of Acupuncture for Migraine Prophylaxis: A Randomized Clinical Trial.” JAMA Internal Medicine 177, no. 4: 4. 10.1001/jamainternmed.2016.9378. 28241154

[brb370536-bib-0059] Zhao, L. , J. Liu , F. Zhang , et al. 2014. “Effects of Long‐Term Acupuncture Treatment on Resting‐state Brain Activity in Migraine Patients: A Randomized Controlled Trial on Active Acupoints and Inactive Acupoints.” PLoS ONE 9, no. 6: e99538. 10.1371/journal.pone.0099538. 24915066 PMC4051855

[brb370536-bib-0060] Zhe, X. , H. Zhang , M. Tang , X. Lei , X. Zhang , and C. Jin . 2023. “Brain Functional Connectivity Patterns Associated With Symptoms of Vestibular Migraine.” Frontiers in Neuroscience 17: 1231273. 10.3389/fnins.2023.1231273. 38156263 PMC10753008

[brb370536-bib-0061] Zhu, J. , R. Gu , L. Shi , and Y. Su . 2024. “Altered Intrinsic Brain Activity in Patients With Neuropathic Pain After Brachial Plexus Avulsion.” Brain Research Bulletin 206: 110831. 10.1016/j.brainresbull.2023.110831. 38056510

[brb370536-bib-0062] Zou, Q.‐H. , C.‐Z. Zhu , Y. Yang , et al. 2008. “An Improved Approach to Detection of Amplitude of Low‐Frequency Fluctuation (ALFF) for Resting‐State fMRI: Fractional ALFF.” Journal of Neuroscience Methods 172, no. 1: 137–141. 10.1016/j.jneumeth.2008.04.012. 18501969 PMC3902859

[brb370536-bib-0063] Zuo, X.‐N. , A. Di Martino , C. Kelly , et al. 2010. “The Oscillating Brain: Complex and Reliable.” Neuroimage 49, no. 2: 1432–1445. 10.1016/j.neuroimage.2009.09.037. 19782143 PMC2856476

[brb370536-bib-0064] Zuo, X.‐N. , R. Ehmke , M. Mennes , et al. 2012. “Network Centrality in the human Functional Connectome.” Cerebral Cortex 22, no. 8: 1862–1875. 10.1093/cercor/bhr269. 21968567

