# Supplementary material for: Multi‐Spatial Voxel‐Scale Modulation of Acupuncture on Abnormal Brain Activity in Migraine Patients Without Aura: A Randomized Study Neuroimaging Trial
Source: Brain Behav. 2025 May 8;15(5):e70536. doi: 10.1002/brb3.70536 (PMC12060218; doi:10.1002/brb3.70536)
Supplement: Supplementary file 1 — Supplementary FIGURE 1. Subgroup analysis of clinical outcome measures in patients with ibuprofen use. Supplementary FIGURE 2. Subgroup analyses of resting state for group and ibuprofen use interactions (p interaction < 0.05, GRF corrected). Supplementary TABLE 1. Subgroup analyses of resting state for group and ibuprofen use interactions. [file BRB3-15-e70536-s001.docx]

**Supplementary FIGURE 1. Subgroup analysis of clinical outcome measures in patients with ibuprofen use**

Abbreviation: CI, confidence interval; HIT-6, Headache Impact Test-6; MSQ-RR: Migraine-Specific Quality of Life Questionnaire, Role Restrictive domain; MSQ-RP: Migraine-Specific Quality of Life Questionnaire, Role Preventive domain; MSQ-EF: Migraine-Specific Quality of Life Questionnaire, Emotional domain; SA: sham acupuncture; TA: true acupuncture; VAS, visual analog scale.

Annotation: Subgroup regression coefficient estimates are represented by squares, with lines through them correspond to the 95% CIs.

**Supplementary FIGURE 2. Subgroup analyses of resting state for group and ibuprofen use interactions (*p*** **interaction < 0.05, GRF corrected).**

**
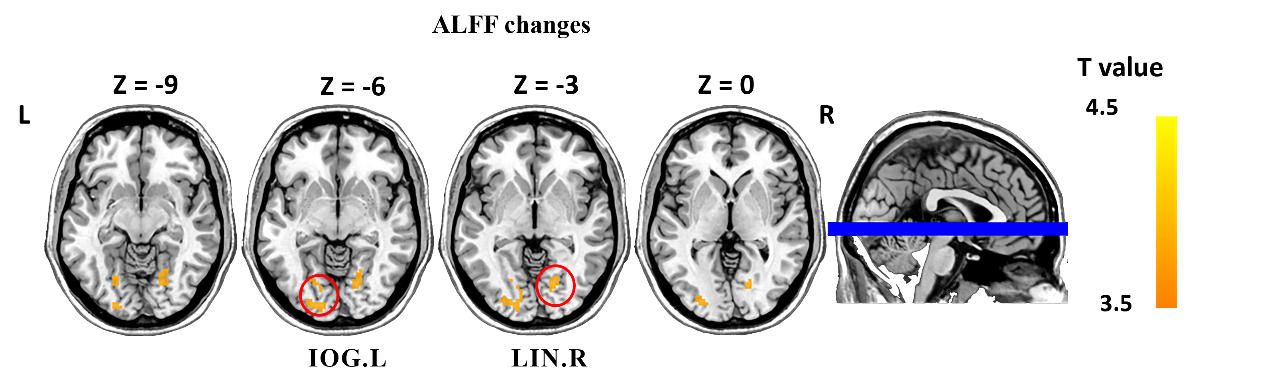
**

Abbreviation: ALFF: frequency fluctuation; IOG: inferior occipital gyrus; L: left; LING: lingual gyrus; R: right.

Notes: Brain regions with altered ALFF values in group-ibuprofen interaction. Warm colors indicate increases.

**Supplementary Table 1 Subgroup analyses of resting state for group and ibuprofen use interactions.**

| **Brain region** | **Hemi** | **MNI coordinate** | | | | **Voxels** | **Change** | **T value** |
| --- | --- | --- | --- | --- | --- | --- | --- | --- |
|  |  | **x** | **y** | | **z** |  |  |  |
| ALFF |  |  |  | |  |  |  |  |
| Lingual | R | 18 | | -69 | -3 | 52 | ↑ | 29.22 |
| Inferior occipital gyrus | L | -24 | | -90 | -6 | 61 | ↑ | 19.52 |

Abbreviation: ALFF, amplitude of low frequency fluctuations; Hemi, hemisphere; MNI, montreal neurological institute.

Annotation: GRF corrected, *p* interaction < 0.05; ↑, increase.
